# Cardiometabolic risk profiles in a Sri Lankan twin and singleton sample

**DOI:** 10.1371/journal.pone.0276647

**Published:** 2022-11-07

**Authors:** Lisa Harber-Aschan, Ioannis Bakolis, Nicholas Glozier, Khalida Ismail, Kaushalya Jayaweera, Gayani Pannala, Carmine Pariante, Fruhling Rijsdijk, Sisira Siribaddana, Athula Sumathipala, Helena M. S. Zavos, Patricia Zunszain, Matthew Hotopf

**Affiliations:** 1 Department of Psychological Medicine, Institute of Psychiatry, Psychology, and Neuroscience, King’s College London, London, United Kingdom; 2 Department Sociology, Stockholm University, Stockholm, Sweden; 3 Department of Biostatistics and Health Informatics, Department of Health Services and Population Research, Institute of Psychiatry Psychology and Neuroscience, King’s College London, London, United Kingdom; 4 Health Services and Population Research Department, Centre for Implementation Science, Institute of Psychiatry Psychology and Neuroscience, King’s College London, London, United Kingdom; 5 Brain and Mind Centre, University of Sydney, Sydney, Australia; 6 Institute for Research and Development, Colombo, Sri Lanka; 7 Stress, Psychiatry and Immunology Laboratory, Institute of Psychiatry, Psychology & Neuroscience, King’s College London, London, United Kingdom; 8 Social Genetic and Developmental Research Centre, Institute of Psychiatry, Psychology & Neuroscience, King’s College London, London, United Kingdom; 9 Department of Medicine, Faculty of Medicine & Allied Health Sciences, Rajarata University of Sri Lanka, Mihintale, Sri Lanka; 10 Research Institute for Primary Care & Health Sciences, Faculty of Medicine & Health Sciences, Keele University, Newcastle-under-Lyme, United Kingdom; 11 Department of Psychology, Institute of Psychiatry, Psychology & Neuroscience, King’s College London, London, United Kingdom; 12 South London and Maudsley NHS Foundation Trust, London, United Kingdom; Tongji Med College, HUST, CHINA

## Abstract

**Introduction:**

Prevention of cardiovascular disease and diabetes is a priority in low- and middle-income countries, especially in South Asia where these are leading causes of morbidity and mortality. The metabolic syndrome is a tool to identify cardiometabolic risk, but the validity of the metabolic syndrome as a clinical construct is debated. This study tested the existence of the metabolic syndrome, explored alternative cardiometabolic risk characterisations, and examined genetic and environmental factors in a South Asian population sample.

**Methods:**

Data came from the Colombo Twin and Singleton follow-up Study, which recruited twins and singletons in Colombo, Sri Lanka, in 2012–2015 (*n* = 3476). Latent class analysis tested the clustering of metabolic syndrome indicators (waist circumference, high-density lipoprotein cholesterol, triglycerides, blood pressure, fasting plasma glucose, medications, and diabetes). Regression analyses tested cross-sectional associations between the identified latent cardiometabolic classes and sociodemographic covariates and health behaviours. Structural equation modelling estimated genetic and environmental contributions to cardiometabolic risk profiles. All analyses were stratified by sex (*n* = 1509 men, *n* = 1967 women).

**Results:**

Three classes were identified in men: 1) “Healthy” (52.3%), 2) “Central obesity, high triglycerides, high fasting plasma glucose” (40.2%), and 3) “Central obesity, high triglycerides, diabetes” (7.6%). Four classes were identified in women: 1) “Healthy” (53.2%), 2) “Very high central obesity, low high-density lipoprotein cholesterol, raised fasting plasma glucose” (32.8%), 3) “Very high central obesity, diabetes” (7.2%) and 4) “Central obesity, hypertension, raised fasting plasma glucose” (6.8%). Older age in men and women, and high socioeconomic status in men, was associated with cardiometabolic risk classes, compared to the “Healthy” classes. In men, individual differences in cardiometabolic class membership were due to environmental effects. In women, genetic differences predicted class membership.

**Conclusion:**

The findings did not support the metabolic syndrome construct. Instead, distinct clinical profiles were identified for men and women, suggesting different aetiological pathways.

## Introduction

As cardiometabolic diseases become increasingly prominent public health challenges in low-and middle-income countries, there is an escalating need to understand variations in cardiometabolic risk in these settings. This is particularly true for South Asian populations where the morbidity of diabetes and cardiovascular is steadily increasing, following rapid urbanisation and Westernisation [1]. In Sri Lanka specifically, cardiovascular diseases and diabetes are the leading causes of mortality in the adult population, together accounting for over a third of all deaths [2, 3]. Identifying reliable and population-specific cardiometabolic risk factors in Sri Lanka and other South Asian countries is therefore necessary for the prevention of these conditions [4].

The metabolic syndrome is a clinical construct of risk factors for cardiometabolic diseases, including central obesity, a poor lipid profile, high blood pressure, and hyperglycemia [5] Whilst some studies find that the metabolic syndrome increases cardiovascular disease risk, beyond its component parts [6–8] the relative importance of the metabolic syndrome components, their measurement, and thresholds cut-offs are debated [9]. More fundamental questions regarding the syndrome’s aetiology, clinical utility, and validity, are also discussed. Studies testing the construct validity of the metabolic syndrome using statistical clustering methods have produced inconsistent results [10]. Whilst some have found support for a cohesive metabolic syndrome construct, [11–13] most find 3–4 clusters, and the clinical composition often varies substantially between ethnicities, countries, gender, and age groups [10, 14–21]. This speaks against the “natural” clustering of components, united by a single underlying mechanism [5], and the universality of the metabolic syndrome. Rather, it suggests that cardiometabolic risk profiles are specific to gender, age and culture, with potentially different aetiological pathways.

As a postulated precursor of cardiometabolic conditions, validating the metabolic syndrome in a South Asian population could provide a tool to identify important risk groups. Understanding the aetiology of cardiometabolic risk in South Asian populations also warrants estimations of genetic contributions, particularly given the “thrifty” genotype hypothesis proposing extreme sensitivity to metabolic dysregulation during obesogenic challenges in these populations [22]. This study used a Sri Lankan population study with a genetically sensitive design to: 1) statistically test the metabolic syndrome; 2) explore alternative characterisations of cardiometabolic risk, and 3) examine the relative influence of genetic and environmental factors contributing to cardiometabolic risk profiles. We hypothesised that a cohesive metabolic syndrome latent class would emerge with elevated cardiometabolic risk for all metabolic syndrome components.

## Methods

### Sample

A representative population sample from the Colombo district, Sri Lanka, was used (the Second Colombo Twin and Singleton Study, CoTaSS-2). CoTaSS-2 is a two-wave cohort study of mental and physical health of twins and singletons, where baseline and follow-up took place in 2007 (N = 5935), and 2012–2015 (N = 3969), respectively (participation rate 76.4%) [23]. Cross-sectional data with complete clinical information from biosamples from the second wave was used (N = 3476). Written informed consent was obtained for each study component that participants opted to partake in. A flowchart of the recruitment procedure, including reports on loss to follow-up is available in Jayaweera et al. [23]. Ethical approval was obtained from the Faculty of Medical Sciences University of Sri Jayewardenepura Ethical Review Committee (USJP ERC, 596/11), and the Psychiatry, Nursing & Midwifery Research Ethics Subcommittee, King’s College London, UK (PNM/10/11-124). This research was funded in whole, or in part, by the Wellcome Trust [Grant number 093206/Z/10/Z]. For the purpose of open access, the author has applied a CC BY-ND public copyright licence to any Author Accepted Manuscript version arising from this submission.

### Procedures

The study consisted of three components: a questionnaire, collection of anthropometric measures, and biosamples collection. The interviews were conducted in English or Sinhala, based on the participant’s preference. Translations of the questionnaires underwent several checks (e.g. multiple independent translations, reviewing translations by an expert panel, and tested by volunteers). Field research assistants received extensive training, and followed structured protocols during the interviews. Quality checks of the data were also performed to ensure reliability. Anthropometric measures were collected by field research assistants at the same time as the interview. Biosample collections took place during early morning visits and included fasting blood samples and first morning urine. These procedures have been described in greater detail elsewhere [23, 24].

### Measures

#### Metabolic syndrome

Metabolic syndrome was calculated according to the International Diabetes Federation and National Cholesterol Education Programme Adult Treatment Panel definitions [25, 26]. The International Diabetes Federation criteria requires central obesity (South Asian-specific waist circumference of ≥90cm for men and ≥80cm for women) and at least two additional components: 1) elevated blood pressure (systolic≥130 mmHg and/or diastolic≥85 mmHg, or hypertensive treatment), 2) elevated plasma triglycerides (≥1.70 mmol/L), 3) low high-density lipoprotein (HDL) cholesterol (<1.03 mmol/L for men, <1.29 mmol/L for women), and 4) elevated fasting plasma glucose (≥5.6 mmol/L or diabetes treatment). The revised National Cholesterol Education Programme Adult Treatment Panel criteria accepts any three components, without holding central obesity as essential. To allow for comparisons between the two definitions, metabolic syndrome was calculated using both approaches. Waist circumference was measured to the nearest 0.1 cm with a measuring tape. Systolic and diastolic blood pressure measures were derived from the mean of three recordings with rest intervals in between. Fasting blood samples provided measures of triglycerides, HDL cholesterol, and fasting plasma glucose. Diabetes was captured from self-reported diabetes diagnoses or medication. Hypertension, heart conditions, hypertensive medication, and cholesterol medication were self-reported. Additional indicators of cardiometabolic risk were obtained from anthropometric measures (Body Mass Index (BMI), based on weight and standing height measured to the nearest kg or 0.1 cm, respectively, and calculated by kg/m^2^), blood samples (total cholesterol (mmol/L), ratio of total cholesterol to HDL cholesterol, low-density lipoprotein cholesterol (mmol/L), very low-density lipoprotein cholesterol (mmol/L), HbA1c (%), insulin (pmol/L), serum glutamic-oxaloacetic transaminase (U/L), and serum creatinine (mg/dL)), and urine samples (microalbumin (mg/L), urine albumin to creatinine ratio, and C-reactive protein (mg/L)) [23]. The non-computerised calculation of the Homeostasis Model Assessment score estimated insulin resistance [27, 28].

#### Socio-demographic measures

A questionnaire adapted from the Sri Lankan census measured sex, age, marital status (married vs. not married), ethnicity (Sinhala vs. Tamil/Muslim/Other), urbanicity (Urban vs. rural/mixed/outside Colombo), education, and occupational class. The education measure captured seven levels of education: no education, Grade 1 to 5, Grade 6 up to O-Levels, passed O-Levels, A-levels, University or higher, and Other. This measure was recoded into three categories of ≤Grade 5, Grade 6-O Levels, and ≥A-Levels. The occupational class measure was derived from an item asking participants about the type of work that they do. The responses were regrouped into the following categories: Managers/Professionals, Skilled manual/non-manual workers (Technicians and associate professionals, Clerks/service workers, Skilled manual workers, Armed forces), Elementary occupations, and Not in employment. Perceived financial strain was measured on a five-point scale and was grouped into Low (living comfortably/doing alright), Moderate (just about getting by), and High (difficult/very difficult to make ends meet). The scale has extensively been used in previous research (e.g. [29, 30]) and draws on measures of perceived financial situation originally conceptualised by Kahn and Pearlin [31].

#### Health behaviours

Physical activity was measured over the past seven days using the International Physical Activity Scale [32]. This is an internationally validated screen, which measures a range of physical activities, and produces a score based on Metabolic Energy Turnover estimated using the time spent on the activity in minutes and the reported number of days the activity was performed. The score was then categorised into Low, Moderate, and High physical activity. A culturally adapted version of a food group frequency questionnaire was used to assess diet [33]. The questionnaire asked about items from 14 food groups and how frequently they had been consumed per day, week, or month. The scale has been validated against dietary recalls and an extended version of the scale [34]. A continuous diet risk variable was constructed by adding binary variables indicating under-consumption of fruit (<2/day) and vegetables (<3/day), and over-consumption deep fried foods, salty snacks, and desserts (≥4/week or daily), producing a score ranging from 0–5. While the diet risk variable has not been validated against other dietary measures, it has face validity given that the individual items are established risk or protective factors of cardiometabolic diseases. The World Health Organization’s Alcohol Use Disorder Identification Test (AUDIT), an internationally validated screen for hazardous or harmful alcohol use, captured alcohol consumption [35]. The AUDIT consists of 10 items which produce a total score ranging from 0–40, where scores ≥8 and ≥16 indicate hazardous and harmful alcohol consumption, respectively. Tobacco smoking was self-reported using the tobacco use questionnaire of the WHO STEPS Instrument, and distinguished between current daily smokers, ex/occasional smokers and those who had never smoked [36].

### Statistical analyses

Latent class analysis was performed in MPlus 7, and descriptive and inferential statistics were performed in Stata 14 [37, 38]. For the purposes of identifying the cardiometabolic classes, the sample was treated as a population sample, using the Stata command *svy* to account for clustering within twin-pairs, and the MPlus *type = complex* command to compute robust standard errors. Analyses were stratified by sex. Latent class analysis addressed the aims (1) to statistically test the metabolic syndrome, and (2) to explore alternative characterisations of cardiometabolic risk. Latent class analysis is a data-driven approach which examines the clustering of individuals, based on their observed continuous or categorical characteristics. It also considers variation in continuous indicators, in contrast to metabolic syndrome combination approaches which uses pre-determined cut-offs. Latent class analysis therefore simultaneously allows for testing the metabolic syndrome and investigating alternative cardiometabolic clusters. The indicators contributing to the latent class analysis included continuous measures of: waist circumference, diastolic blood pressure, systolic blood pressure, fasting plasma glucose, HDL cholesterol and triglycerides; and binary variables of: hypertension medication, cholesterol medication, and diabetes/diabetes medication. Error terms for diastolic and systolic blood pressure were correlated to account for violation of the independence assumption. Triglyceride and fasting plasma glucose measures were log-transformed. Age was entered as an auxiliary variable. Maximum-likelihood methods estimated the model parameters.

Consistent with conventional latent class analysis practice we forced a 2-class solution, and then re-estimated the model with 3, 4 and 5 classes. The model solutions were compared using statistical model fit indices as well as clinical and theoretical interpretability to select the best model. Individuals were then grouped into class memberships based on their highest posterior probability estimates. Model fit indices included the: Akaike Information Criteria [39], Bayesian Information Criteria [40], Sample size Adjusted Bayesian Information Criteria [41], entropy [42], and Lo–Mendell–Rubin likelihood ratio test [43]. Descriptive statistics estimated means and percentages with 95% confidence intervals (CI) to describe classes according to clinical variables contributing to the model, relevant clinical measures, socio-demographic and socio-economic characteristics and health behaviours. Unadjusted and adjusted multinomial regression analysis explored associations between the latent classes and socio-demographic characteristics, estimating relative risk ratios (RRR) and 95% CI. The regression models applied listwise deletion to handle missing data. Alcohol and tobacco use were only examined in men, as these were rare among women (<1%) [23].

#### Twin analysis

Aim (3) to examine the relative influence of genetic and environmental factors contributing to cardiometabolic risk profiles, was addressed with structural equation twin model-fitting. Dichotomous variables were generated to address the nominal class structure. A liability threshold model was fitted to each dichotomous class variable for men and women. This model assumes the observed proportions of e.g., variable ‘1 not 1’ to reflect an underlying normally distributed liability that represents an individual’s propensity of belonging to that class, with the threshold indicating the prevalence of the class. A pair’s joint distribution is similarly represented by a bivariate normal distribution. Differential tetrachoric correlations in monozygotic and dizygotic twin pairs provided the power to decompose the variance of the latent liability of class membership into three independent sources: additive genetic, shared environment which makes family members similar, and non-shared unique environment, including measurement error, which makes family members different [44]. Model-fitting was conducted in OpenMx with raw data maximum likelihood estimation [45]. Since the classes were qualitatively different across men and women, opposite-sex pairs could not be used, however, singletons were included to inform the threshold (and the effect of age on this threshold) representing the prevalence of the classes.

## Results

The mean age of men was 42.0 (N = 1509), and 43.3 in women (N = 1967; Table 1). Similar proportions of men and women were married (73%) and of Sinhala ethnicity (93%). Most lived in urban areas and had at least some secondary education.

**Table 1:** Descriptive table and latent cardiometabolic classes.

	Men N = 1509		Women N = 1967	
Twins and singletons				
Twins	1160	76.9	1417	72.0
Singletons	349	23.1	550	28.0
Age (years)	1,509	42.0 (14.5)	1,967	43.3 (14.2)
Age categories				
19–34	557	36.9	611	31.1
35–49	502	33.3	723	36.8
50–59	235	15.6	361	18.4
≥60	215	14.2	272	13.8
Marital status				
Married	1,096	73.5	1,417	72.6
Not married	395	26.5	536	27.4
Ethnicity				
Sinhala	1,391	93.4	1,806	92.5
Tamil/Muslim/Other	99	6.6	147	7.5
Urbanicity				
Urban	893	59.2	1,183	60.1
Rural, mixed, or outside Colombo	616	40.8	1,183	39.9
Education				
≤ Grade 5	97	6.5	178	9.2
Grade 6—O/Ls	947	63.9	1,179	60.6
≥ A/Ls	439	29.6	587	30.2
Occupational class				
Managers/Professionals	136	9.2	130	6.7
Skilled manual/non-manual workers	902	60.9	481	24.8
Elementary occupations	193	13.0	95	4.9
Not in employment	250	16.9	1,233	63.6
Financial strain				
Low	1,172	78.6	1,418	72.6
Moderate	192	12.9	293	15.0
High	127	8.5	241	12.3
Latent cardiometabolic classes in men				
Class 1: Healthy (Healthy WC, Healthy TG, Healthy HDL-C, Healthy BP Healthy FPG)	798	52.3		
Class 2: WC, TG, FPG (High WC, High TG, Healthy HDL-C, Healthy BP, Raised FPG)	606	40.2		
Class 3: WC, TG, Diabetes (High WC, High TG, Healthy HDL-C, Healthy BP, Very high levels of FPG)	114	7.6		
Latent cardiometabolic classes in women				
Class 1: Healthy, WC (High WC, Healthy TG, Healthy HDL-C, Healthy BP, Healthy FPG)			1,046	54.2
Class 2: Obese, HDL-C, Treated BP, FPG (Very high WC, Borderline raised TG, Low HDL-C, Healthy BP but BP medication, Raised FPG)			646	32.8
Class 3: WC, Diabetes (Very high WC, Borderline raised TG, Healthy HDL-C, Borderline raised BP, Very high levels of FPG)			142	7.2
Class 4: WC, Untreated BP, FPG (High WC, Healthy TG, Healthy HDL-C, High BP but no BP medication, Raised FPG)			133	6.8

BP, blood pressure; FPG, fasting plasma glucose; HDL-C, high density lipoprotein cholesterol; TG, triglyceride; WC, waist circumference.

Healthy values for each of the components are: WC <90.0 cm for men and <80.0 cm for women, TG <1.7 mmol/L, HDL-C >1.03 mmol/L, systolic/diastolic BP <130.0/85.0 mm Hg, FPG <5.6 mmol/L.

“Raised” levels refer to values that are close to, but over, the healthy threshold and where the threshold is not included in within the 95% confidence intervals for the mean of the class.

“Borderline raised” levels refer to values that are close to the healthy threshold, and the where threshold is included within the 95% confidence intervals for the mean of the class.

“Very high levels” refer to values that are substantially elevated above the healthy threshold.

FPG values ≥7.0 mmol/l are labelled as diabetes (Class 3 in men and women).

Statistics for continuous variables present n and mean ± standard deviation (SD), while categorical variables present n and %.

Numbers may not add up due to missing data.

Guided by model fit statistics and interpretability, we opted for the 3-class solution in men, and the 4-class solution in women (see S1 Table for fit statistics). In men, the Akaike Information Criteria, Bayesian Information Criteria and Sample size adjusted Bayesian Information Criteria improved with additional classes, but the Lo–Mendell–Rubin likelihood ratio test indicated that no further model fit improvements were gained beyond 3 classes. Entropy dropped from 2 to 3 classes, suggesting poorer parsimony, but remained acceptable. Despite poorer parsimony, we opted for the 3-class solution as the classes were more clinically meaningful upon inspection. In women, the Akaike Information Criteria, Bayesian Information Criteria and Sample size adjusted Bayesian Information Criteria improved with additional classes until the 4-class solution, and all models had good entropy (>0.8) indicating clearly delineated classes.

**Fig 1:**
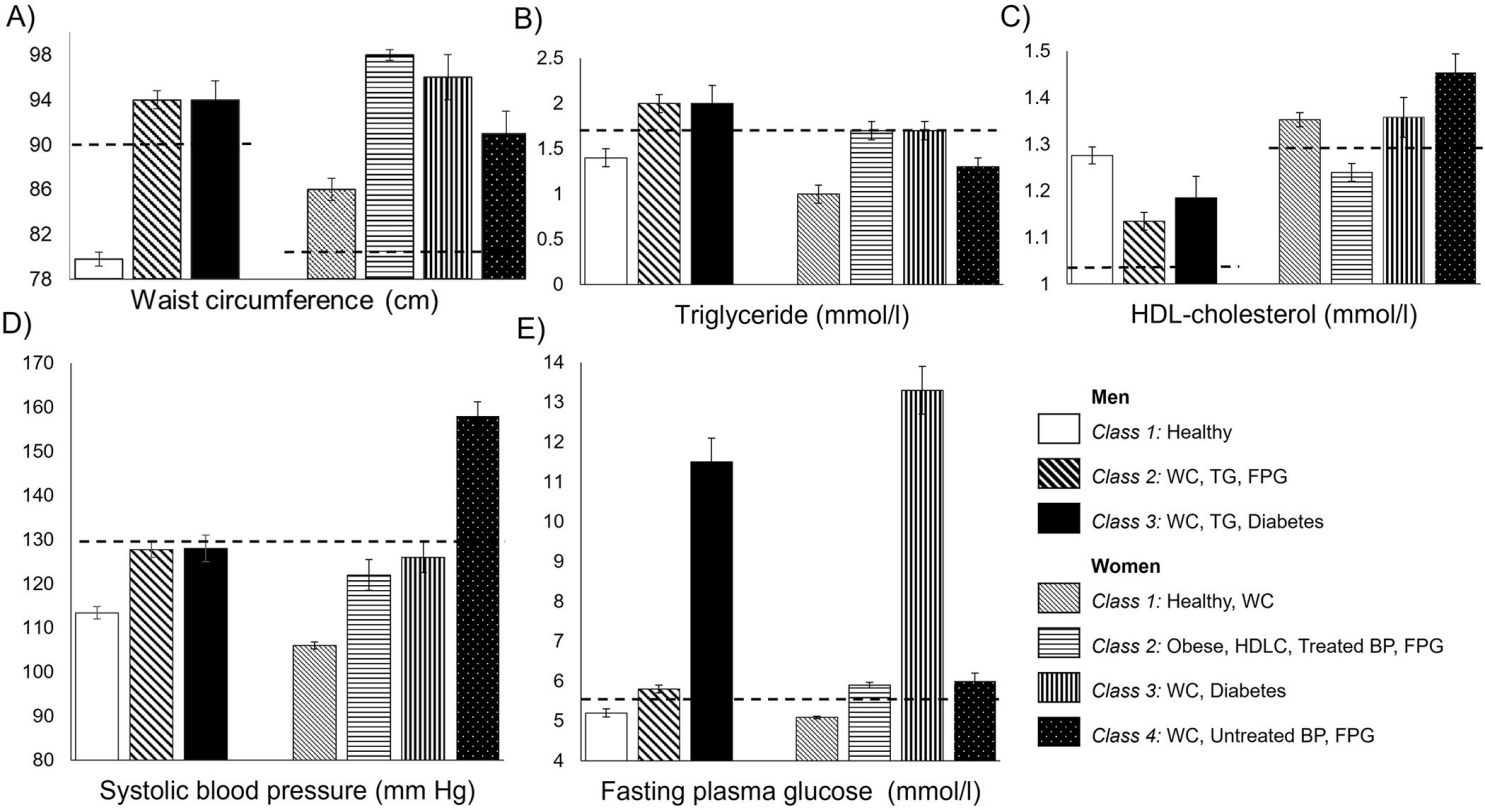
Cardiometabolic classes by metabolic syndrome components. A) Waist circumference (cm), B) Triglyceride (mmol/l), C) HDL cholesterol, D) Systolic blood pressure (mm Hg), and E) Fasting plasma glucose (mmol/l), in male (N = 1509) and female (N = 1967) CoTaSS-2 participants in 2012–2015, Colombo, Sri Lanka. The dashed horizontal lines indicate risk thresholds according to the International Diabetes Federation and National Cholesterol Education Programme Adult Treatment Panel criteria. Higher values indicate poorer cardiometabolic function with the exception of HDL cholesterol where values below the line indicate poor cardiometabolic health. Diastolic blood pressure distributions were comparable to the systolic blood pressure presented in D) (not shown). BP, blood pressure; FPG, fasting plasma glucose; HDL-C, high-density lipoprotein cholesterol; TG, triglyceride, WC, Waist circumference.

Fig 1A–1E presents the latent classes by metabolic syndrome components in men and women (see S2 and S3 Tables for exact means). 52% of men were in Class 1, a “Healthy” class; 40.2% were in Class 2 characterised by high waist circumference, high triglycerides and raised fasting plasma glucose; and 7.6% in Class 3 had high waist circumference and triglycerides, and substantially raised levels of fasting plasma glucose. Men in Classes 2 and 3 were similar on most metabolic syndrome components, but Class 2 had fasting plasma glucose levels just above threshold (5.8 mmol/L), while men in Class 3 had nearly twice as high fasting plasma glucose levels (11.5 mmol/L).

54% of women belonged to a “Healthy” class (Class 1), with waist circumference measures above threshold (85.6 cm vs. the risk threshold of 80.0 cm according to the International Diabetes Federation and National Cholesterol Education Programme Adult Treatment Panel criteria, Fig 1), but an otherwise healthy cardiometabolic profile. Low HDL cholesterol specifically affected women in Class 2 (32.8%), who also had raised triglycerides and fasting plasma glucose. Whilst women in Class 2 had the lowest blood pressure levels, it seems this reflected well-managed hypertension, given that a high proportion reported blood pressure medication (S3 Table). Substantially raised fasting plasma glucose levels were characteristic for women in Class 3 (7.2%), while untreated hypertension was a distinct characteristic of women in Class 4 (6.8%). The mean waist circumference was above the risk threshold for all female Classes, but particularly high in Class 2.

Class 1, the “Healthy” class, and Class 3, the “Diabetes” class, were comparable in their clinical profiles and proportions between men and women. There were also notable gender differences. Women’s waist circumference in all classes exceeded the sex-specific cut-off more than for men. Triglyceride levels were above the risk threshold in male Classes 2 and 3, whilst only borderline threshold for women in Classes 2 and 3. None of the male classes were characterised by poor HDL cholesterol profiles or high blood pressure, while this specifically affected women in Class 2 and Class 4, respectively.

Table 2 presents adjusted associations between socio-demographic characteristics and health behaviours with the cardiometabolic classes, compared to Class 1 (S4 and S5 Tables present prevalence distributions and unadjusted analyses). In men, there was strong evidence that older age and being married increased the risk of membership in Class 2 and 3, compared to Class 1. Men with no educational qualifications and in elementary occupations were at substantially lower risk of placement in Classes 2 and 3, relative to the “Healthy” Class 1. U-shaped trends were observed for physical activity and alcohol use in men, such that moderate—but not low—physical activity, and hazardous—but not harmful—alcohol use was associated with risk of placement in Class 2 and 3, relative to the reference. In women, there was strong evidence for age being a determinant of cardiometabolic class membership; older age being particularly strongly associated with placement in Class 4. For women, education, occupational class, financial strain, physical activity, and diet did not determine class membership. Adjusted regression analyses exploring differences between the cardiometabolic classes, indicated that age was the only covariate which distinguished classes from each other. In men, older age was associated with increased risk of membership in Class 3 over Class 2 (RRR = 1.04, 95% CI: 1.02, 1.06). Similarly, in women, older age was associated with increasing risk of membership across the classes (Class 3 relative to Class 2: RRR = 1.02, 95% CI: 1.00, 1.03; Class 4 relative to Class 2: RRR = 1.12, 95% CI: 1.09, 1.15; Class 4 relative to Class 3: RRR = 1.10, 95% CI: 1.08, 1.14; analyses not shown).

**Table 2:** Adjusted^a^ associations between latent cardiometabolic classes and socio-demographic characteristics and health behaviorus.

	Men (n = 1509)					Women (n = 1967)						
	Class 1 Healthy (52.3%)	Class 2 WC, TG, FPG (40.2%)		Class 3 WC, TG, Diabetes (7.6%)		Class 1 Healthy, WC (53.1%)	Class 2 Obese, HDL-C, Treated BP, FPG (32.8%)		Class 3 WC, Diabetes (7.2%)		Class 4 WC, Untreated BP, FPG (6.8%)	
	RRR	RRR	(95% CI)	RRR	(95% CI)	RRR	RRR	(95% CI)	RRR	(95% CI)	RRR	(95% CI)
Age (years)	1.00	1.04	(1.02 to 1.05)	1.07	(1.05 to 1.09)	1.00	1.09	(1.08 to 1.11)	1.11	(1.09 to 1.13)	1.23	(1.20 to 1.26)
Not married (vs. married)	1.00	0.50	(0.36 to 0.70)	0.31	(0.14 to 0.67)	1.00	0.58	(0.45 to 0.77)	0.38	(0.22 to 0.64)	0.63	(0.35 to 1.11)
Ethnic minority (vs. Sinhala)	1.00	1.22	(0.75 to 2.00)	2.02	(0.93 to 4.38)	1.00	1.40	(0.90 to 2.16)	1.83	(0.90 to 3.69)	0.79	(0.24 to 2.56)
Education												
≤ Grade 5	1.00	0.39	(0.22 to 0.69)	0.27	(0.09 to 0.81)	1.00	0.95	(0.57 to 1.59)	0.60	(0.24 to 1.51)	0.78	(0.30 to 2.01)
Grade 6—O/Ls	1.00	0.67	(0.50 to 0.91)	0.83	(0.47 to 1.47)	1.00	1.03	(0.78 to 1.36)	1.13	(0.69 to 1.85)	1.65	(0.88 to 3.12)
≥ A/Ls		1.00		1.00			1.00		1.00		1.00	
Occupational class												
Managers/Professionals		1.00		1.00			1.00		1.00		1.00	
Skilled manual/non-manual workers	1.00	1.02	(0.64 to 1.63)	0.57	(0.26 to 1.22)	1.00	0.92	(0.54 to 1.57)	0.84	(0.34 to 2.12)	0.39	(0.10 to 1.63)
Elementary occupations	1.00	0.51	(0.28 to 0.93)	0.23	(0.08 to 0.68)	1.00	0.60	(0.29 to 1.25)	0.89	(0.28 to 2.78)	0.42	(0.09 to 2.03)
Not in employment	1.00	1.26	(0.72 to 2.20)	0.51	(0.20 to 1.31)	1.00	0.97	(0.58 to 1.65)	0.78	(0.32 to 1.92)	0.61	(0.18 to 2.10)
Financial strain												
Low		1.00		1.00			1.00		1.00		1.00	
Moderate	1.00	0.61	(0.42 to 0.88)	0.74	(0.37 to 1.45)	1.00	0.99	(0.72 to 1.36)	1.21	(0.72 to 2.03)	1.19	(0.63 to 2.23)
High	1.00	1.04	(0.68 to 1.60)	1.25	(0.61 to 2.58)	1.00	0.97	(0.67 to 1.39)	1.36	(0.77 to 2.40)	1.25	(0.64 to 2.44)
Physical activity												
Low	1.00	1.20	(0.77 to 1.88)	1.60	(0.74 to 3.48)	1.00	0.87	(0.55 to 1.37)	1.39	(0.69 to 2.80)	1.26	(0.48 to 3.32)
Moderate	1.00	1.71	(1.36 to 2.47)	2.15	(1.28 to 3.61)	1.00	0.99	(0.74 to 1.30)	1.00	(0.61 to 1.64)	0.94	(0.53 to 1.66)
High		1.00		1.00			1.00		1.00		1.00	
Diet risk^b^	1.00	1.08	(0.97 to 1.21)	1.11	(0.88 to 1.38)	1.00	1.05	(0.93 to 1.17)	0.98	(0.81 to 1.18)	1.01	(0.80 to 1.27)
Alcohol use												
No misuse		1.00		1.00								
Hazardous use	1.00	1.83	(1.36 to 2.47)	2.00	(1.15 to 3.47)							
Harmful use	1.00	0.98	(0.59 to 1.64)	1.93	(0.85 to 4.35)							
Smoking												
Never smoked		1.00		1.00								
Ex/occasional smoker	1.00	1.10	(0.80 to 1.50)	0.83	(0.47 to 1.46)							
Current daily smoker	1.00	0.74	(0.53 to 1.03)	0.97	(0.54 to 1.73)							

^a^ Associations are adjusted for all variables presented in the table

^b^ Diet risk score is on a scale from 0–5, where higher scores indicate poorer diet.

RRR, relative risk ratio; BP, blood pressure; FPG, fasting plasma glucose; HDL-C, high density lipoprotein cholesterol; TG, triglyceride; WC, waist circumference.

Approximately half of the men in Class 2 and 58.8–71.9% of men in Class 3 met the metabolic syndrome criteria, while very few men in Class 1 did so (Table 3). In women, metabolic syndrome was prevalent among approximately 10%, 70%, 80% and 65% of women in Classes 1–4, respectively. The distribution of other cardiometabolic risk factors indicated clinical profiles which broadly supported the characterisations of the cardiometabolic classes (S6 and S7 Tables). Men and women in respective Class 1, identified as “healthy”, had healthy BMIs and good overall clinical profiles. Fasting plasma glucose measures were consistent with measures of glycated haemoglobin and insulin resistance, cholesterol measures were broadly consistent with HDL cholesterol levels, and self-reported hypertension was highest in the female Class 4, the untreated hypertension class. Microalbumin, Urine Albumin to Creatinine Ratio and C-reactive protein levels mirrored the fasting plasma glucose levels, being substantially higher among in men and women in Class 3, characterised by diabetes, indicating adverse renal function and inflammation.

**Table 3:** Overlap between latent cardiometabolic classes and metabolic syndrome.

	Men (n = 1509)						Women (n = 1967)							
	Class 1 Healthy (52.3%)		Class 2 WC, TG, FPG (40.2%)		Class 3 WC, TG, Diabetes (7.6%)		Class 1 Healthy, WC (53.1%)		Class 2 Obese, HDL-C, Treated BP, FPG (32.8%)		Class 3 WC, Diabetes (7.2)		Class 4 WC, Untreated BP, FPG (6.8%)	
	%	(95% CI)	%	(95% CI)	%	(95% CI)	%	(95% CI)	%	(95% CI)	%	(95% CI)	%	(95% CI)
MetS (IDF)	0.6	(0.3 to 1.5)	42.4	(38.5 to 46.4)	58.8	(49.0 to 67.9)	9.8	(8.1 to 11.8)	73.4	(69.8 to 76.7)	79.6	(71.8 to 85.7)	65.9	(57.5 to 73.4)
MetS (ATP III)	3.3	(2.3 to 4.8)	51.1	(47.1 to 55.0)	71.9	(62.6 to 79.7)	9.7	(8.0 to 11.7)	73.7	(70.2 to 77.0)	81.0	(73.3 to 86.9)	65.9	(57.4 to 73.5)

BP, blood pressure; FPG, fasting plasma glucose; HDL-C, high density lipoprotein cholesterol; MetS (ATP III), National Cholesterol Education Programme Adult Treatment Panel definition; MetS (IDF), metabolic syndrome, International Diabetes Federation definition; TG, triglyceride; WC, waist circumference.

For the twin analyses, sample size did not permit decomposing class-membership variance for Class 3 in men (N = 114) and Class 4 in women (N = 142) due to few concordant pairs. For the other classes, individual differences in class membership in males was equally due to the two components that make up familial effects: genetic and shared-environmental variance (Table 4). There was, however, no power to establish the significance of these components independently, only their sum (familial effects). For women, in contrast, genetic effects determined membership of all Classes, with high heritability estimates (70–78%). The small dizygotic twin correlations in women suggested fitting a model with non-additive genetic effects rather than shared environment. However, the analysis was under-powered to estimate two different genetic sources, and we primarily aimed to estimate overall heritabilities [44, 45].

**Table 4:** Twin correlations and ACE decomposition of class membership for males and females.

	r_MZ_	(95% CI)	r_DZ_	(95% CI)	h^2^	(95% CI)	c2	(95% CI)	e^2^	(95% CI)
Men (n = 1160)										
Class 1: Healthy	0.76	(0.61 to 0.86)	0.54	(0.30 to 0.73)	0.40	(0.00 to 0.86)	0.40	(0.00 to 0.76)	0.20	(0.11 to 0.32)
Class 2: WC, TG, FPG	0.65	(0.48 to 0.78)	0.42	(0.15 to 0.64)	0.46	(0.00 to 0.80)	0.23	(0.00 to 0.67)	0.31	(0.19 to 0.47)
Women (n = 1600)										
Class 1: Healthy, WC	0.75	(0.61 to 0.85)	0.05	(-0.24 to 0.34)	0.73	(0.35 to 0.92)	0.00	(0.00 to 0.20)	0.28	(0.17 to 0.43)
Class 2: Obese, HDLC, Treated BP, FPG	0.66	(0.50 to 0.79)	0.13	(-0.14 to 0.37)	0.70	(0.39 to 0.81)	0.00	(0.00 to 0.27)	0.30	(0.19 to 0.44)
Class 3: WC, Diabetes	0.73	(0.44 to 0.90)	0.14	(-0.41 to 0.62)	0.78	(0.08 to 0.92)	0.00	(0.00 to 0.61)	0.22	(0.08 to 0.46)

c2, standardised shared-environmental effects; e2 = standardised unique-environmental effects; h2, standardised genetic effects; rDZ = dizygotic twin correlation; rMZ, monozygotic twin correlation

Number of twin types contributing to the models are: monozygotic male twins n = 483, dizygotic male twins n = 325, male triplets n = 3, male singletons n = 349, monozygotic female twins n = 621, dizygotic female twins n = 421, female triplets n = 8, female singletons n = 550.

## Discussion

This study found limited support for a unified metabolic syndrome construct in a Sri Lankan population sample. Model solutions with more than two classes fitted the data better, and none could be characterised as a specific metabolic syndrome class. Instead, we found distinct clinical profiles, specific to men and women, with high cardiometabolic risk on some components, while others within the same class were within healthy ranges or only slightly raised. Specifically, for women, the identified classes had distinct cardiometabolic risk profiles, where Class 3 was characterised by very high fasting plasma glucose, and Class 4 by very high blood pressure. For men, the cardiometabolic risk classes were more comparable across components, but distinguished by substantially raised fasting plasma glucose values in Class 3. Class 2 in women had the closest match to a congruent metabolic syndrome construct, with very high waist circumference, low HDL cholesterol, treated hypertension, and raised triglycerides and fasting plasma glucose. However, substantial heterogeneity across indicators was observed: whilst waist circumference and HDL cholesterol exceeded the threshold substantially, the other components were not as high as would be expected if they were to cluster “naturally”, driven by a single underlying mechanism [5].

Support for different versions of metabolic syndrome classes have been found in other studies using latent class analysis. For example, a two-class solution was found in men and women of Hispanic ethnicity in the US [12]; a study of Japanese-American men found a distinct metabolic syndrome class in a three-class solution [16]; a study of an Iranian population sample observed a metabolic syndrome class among four identified classes [18]; and a US study identified a metabolic syndrome class in an ethnically diverse population sample [19]. However, the identified metabolic syndrome classes did not score highly consistently across all components [18, 19], certain components were excluded for the purpose of achieving parsimony [12], and additional distinct cardiometabolic risk classes were typically found with high risk on some components, consistent with our findings [16, 18, 19]. Furthermore, the identified metabolic syndrome classes varied in size, and in terms of the prominence of metabolic syndrome components contributing to them, and differed by gender [19]. Our results contribute to this heterogenous evidence base, indicating that there is substantial variation in the expression of metabolic syndrome component clustering, and limited evidence for a single underlying metabolic syndrome construct. Research directly comparing metabolic syndrome component clustering between countries in Europe observed substantial cross-cultural variation, supporting this interpretation [17].

Our findings are consistent with past studies using factor analyses of Asian population samples which also identified multiple gender-specific clusters of metabolic syndrome components [14, 15]. A study using a multi-ethnic Asian household survey which oversampled ethnic minorities (N = 1957 men, N = 2308 women) found that hypertension had a weak association with fasting plasma glucose and insulin resistance in Indian women [15], consistent with the female Class 4 in our study, characterised by hypertension and relatively low fasting plasma glucose. Our study extends this evidence base of metabolic syndrome clusters in Asian populations, using a latent class analysis approach which allows for using both binary and continuous variables, thus including those with diabetes and hypertension medication, rather than excluding these individuals as some past studies have done [15]. Our observation that the largest cardiometabolic risk class in women was characterised by low HDL cholesterol is also consistent with a study which found that low HDL cholesterol was particularly prominent in Indian women living in the US (N = 226 men and women) [46].

Low SES was protective of membership in Classes 2 and 3 in men; a finding which is consistent with observations of diabetes and hypertension being more prevalent in higher SES groups in South Asian populations [47, 48] This may suggest that the lifestyle factors of men with higher SES in Sri Lanka are associated with greater cardiometabolic risk [49]. However, this association held whilst adjusting for health behaviours including diet, alcohol consumption, exercise and smoking, suggesting that either unmeasured aspects of high SES in men puts them at increased cardiometabolic risk, or that the lifestyle questions were prone to misclassification. The lack of an association between SES and cardiometabolic risk in women suggests that the potentially harmful influence of men’ high SES environments may not extend to women in Sri Lanka; the difference in lifestyles between men with low and high SES being greater, compared to women. Limited environmental variation may also explain why genetic effects were only observed in women given that it allowed greater opportunity for genetic effects, and why only age distinguished the latent classes in women, potentially reflecting a progression cardiometabolic risk over women’s life course, or alternatively, cohort effects.

### Strengths & limitations

The novelty of the study represents an important strength. It is the first study to test the clustering of metabolic syndrome components in a Sri Lankan population sample, which provides a detailed description of local risk profiles, and is also the first to assess metabolic syndrome component clustering in a large representative population sample with a genetically sensitive design. There are also some limitations that ought to be acknowledged. While the identified cardiometabolic classes provide insight into the clustering of cardiometabolic risks in this sample, these findings ought to be replicated in other datasets before drawing wider conclusions about the classes’ generalisability. Furthermore, our analysis was limited to metabolic syndrome components; future research should consider including other clinical measures to improve cardiometabolic risk characterisation and prediction. The cross-sectional data also represents an important limitation as it is not clear from our study how the identified cardiometabolic classes relate to future risk of cardiometabolic conditions. It also limits the causal inferences made regarding environmental and behavioural risk factors. With regards to potential measurement bias, all respondents could self-report medications, but direct assessment was incomplete for all metabolic syndrome components. Thus, selective medication reporting may have influenced group membership.

### Implications

We hypothesised that a cohesive metabolic syndrome latent class would emerge with elevated cardiometabolic risk for all metabolic syndrome components, indicative of an underlying and unifying metabolic syndrome construct. We found no support for this hypothesis, and the failure to identify a cohesive cluster of cardiometabolic risk components is a finding which has substantial support in the existing literature. This suggests that cardiometabolic risk cannot be universally captured in a construct such as metabolic syndrome, but that cardiometabolic risk is culturally-, ethnicity- and sex-specific with distinct aetiological pathways. Despite the accumulating evidence of heterogeneity in cardiometabolic risk profiles, fundamental questions about the existing definition of metabolic syndrome are rarely raised [10], but the discussions tend to encourage exploration of “metabolic syndrome sub-types” instead [50]. However, it is arguably inappropriate speak of sub-types when the evidence for a unifying metabolic syndrome construct is limited.

Our results may also raise questions regarding the clinical usefulness of metabolic syndrome, given that no class perfectly overlapped with metabolic syndrome. Even for Class 3 which captured 80% of women with metabolic syndrome, this nevertheless leaves 1 in 5 women with alarmingly high fasting plasma glucose levels unidentified by the metabolic syndrome. Simultaneously, metabolic syndrome misclassifies 1 in 10 women with a seemingly healthy cardiometabolic profile (Class 1). While our cross-sectional data does not allow us to compare the predictive ability of metabolic syndrome against the identified cardiometabolic classes for future cardiometabolic risk, it does suggest that the metabolic syndrome might be a blunt tool for capturing cardiometabolic risk which potentially fails to identify a substantial proportion of people who could benefit from clinical intervention, whilst erroneously labelling a substantial minority of healthy women. Indeed, previous studies suggest that metabolic syndrome is imprecise in terms of risk prediction [51], and offers limited additional value beyond its component parts [52, 53]. Other risk prediction tools or, indeed, specific clusters or individual metabolic syndrome components may therefore predict cardiovascular disease and cardiovascular aging more accurately [54, 55].

Whilst cross-sectional data does not permit determining whether the identified classes are superior for capturing cardiometabolic risk over the metabolic syndrome, the results nevertheless bear some immediate clinical implications. Population-specific predictors of cardiometabolic risk are urgently needed in order to meet WHO targets to reduce the burden non-communicable diseases in South Asia [4]. The risk profiles could be meaningful to clinicians, as simple demographic indicators such as age and sex could inform which cardiometabolic risk factors to prioritise in terms of screening and targeting for intervention. For public health purposes, the classes also indicate the greatest local population needs, which is important to inform the upscaling of healthcare services in the region in efforts to address non-communicable diseases [4, 56]. Furthermore, the observation that the waist circumference of women in the “healthy” class was substantially over the advised cut-off suggests that this recommendation for female central obesity may be substantially lower than necessary.

## Conclusion

In this Sri Lankan population sample distinct clinical profiles emerged suggesting that cardiometabolic risk is more complex than a single underlying metabolic syndrome construct.

## Supporting information

S1 Table Model fit statistics for latent class solutions in men and women. (DOCX)

S2 Table Description of latent cardiometabolic classes by metabolic syndrome components contributing to the model in men (N = 1509). (DOCX)

S3 Table Description of latent cardiometabolic classes by metabolic syndrome components contributing to the model in women (N = 1967). (DOCX)

S4 Table Prevalence distribution and unadjusted associations of sociodemographic characteristics and health behaviours with latent classes in men (N = 1509). (DOCX)

S5 Table Prevalence distribution and unadjusted associations of sociodemographic characteristics and health behaviours with latent classes in women (N = 1967). (DOCX)

S6 Table Description of latent cardiometabolic classes according to other cardiometabolic risk factors in men (N = 1509). (DOCX)

S7 Table Description of latent cardiometabolic classes according to other cardiometabolic risk factors in women (N = 1967). (DOCX)
