# Supplementary material for: Cardiometabolic risk profiles in a Sri Lankan twin and singleton sample
Source: PLoS One. 2022 Nov 7;17(11):e0276647. doi: 10.1371/journal.pone.0276647 (PMC9639827; doi:10.1371/journal.pone.0276647)
Supplement: S1 Table — (DOCX) [file pone.0276647.s001.docx]

S1 Table. Model fit statistics for latent class solutions in men and women

| **Model: Number of classes** | **AIC** | **BIC** | **SABIC** | **E** | **LMR-LRT**  ***P* value** |
| --- | --- | --- | --- | --- | --- |
| Men |  |  |  |  |  |
| 2 class | 37,058 | 37,196 | 37,114 | 0.976 | <0.001 |
| 3 class | 36,571 | 36,763 | 36,648 | 0.728 | 0.011 |
| 4 class | 36,274 | 36,519 | 36,373 | 0.746 | 0.162 |
| 5 class | 36,045 | 36,343 | 36,165 | 0.789 | 0.053 |
| Women |  |  |  |  |  |
| 2 class | 49,920 | 50,065 | 49,983 | 0.974 | <0.001 |
| 3 class | 48,913 | 49,114 | 49,000 | 0.853 | 0.012 |
| 4 class | 48,375 | 48,631 | 48,485 | 0.801 | 0.004 |
| 5 class | 47,993 | 48,306 | 48,128 | 0.819 | 0.068 |
| AIC; Akaike Information Criteria; BIC, Bayesian Information Criteria; E, Entropy; LMR-LRT, Lo–Mendell–Rubin Likelihood Ratio Test; SABIC, Sample size Adjusted Bayesian Information Criteria. | | | | | |
|  | | | | | |
