# Supplementary material for: Cardiometabolic risk profiles in a Sri Lankan twin and singleton sample
Source: PLoS One. 2022 Nov 7;17(11):e0276647. doi: 10.1371/journal.pone.0276647 (PMC9639827; doi:10.1371/journal.pone.0276647)
Supplement: S2 Table — (DOCX) [file pone.0276647.s002.docx]

**S2 Table. Description of latent cardiometabolic classes by metabolic syndrome components contributing to the model in men (N=1509)**

|  | **Healthy**  **values** |  | **Class 1**  Healthy (52.3%) | | |  | | **Class 2**  WC, TG, FPG (40.2%) | | |  | **Class 3**  WC, TG, Diabetes (7.6%) | | |
| --- | --- | --- | --- | --- | --- | --- | --- | --- | --- | --- | --- | --- | --- | --- |
|  |  |  | Mean | % | 95% CI |  | Mean | | % | 95% CI |  | Mean | % | 95% CI |
| WC (cm) | <90.0 |  | 79.8 |  | 79.2, 80.4 |  | 94.0 | |  | 93.2, 94.7 |  | 94.0 |  | 92.3, 95.7 |
| TG (mmol/L) | <1.7 |  | 1.4 |  | 1.3, 1.4 |  | 2.0 | |  | 1.9, 2.1 |  | 2.0 |  | 1.8, 2.2 |
| HDL-C (mmol/L) | >1.03 |  | 1.3 |  | 1.3, 1.3 |  | 1.1 | |  | 1.1, 1.2 |  | 1.2 |  | 1.1, 1.2 |
| Systolic BP (mm Hg) | <130.0 |  | 113.4 |  | 112.4, 114.3 |  | 127.7 | |  | 126.3, 129.1 |  | 128.2 |  | 125.2, 131.2 |
| Diastolic BP (mm Hg) | <85.0 |  | 72.7 |  | 72.1, 73.4 |  | 82.5 | |  | 81.6, 83.5 |  | 82.6 |  | 80.7, 84.4 |
| FPG (mmol/L) | <5.6 |  | 5.2 |  | 5.1, 5.2 |  | 5.8 | |  | 5.7, 5.8 |  | 11.5 |  | 10.9, 12.0 |
| Diabetes |  |  |  | 0.1 | 0.0, 0.9 |  |  | | 15.4 | 12.7, 18.7 |  |  | 65.2 | 55.7, 73.6 |
| Blood pressure medication |  |  |  | 0.0 |  |  |  | | 20.1 | 17.0 23.6 |  |  | 24.6 | 17.5, 33.3 |
| Cholesterol medication |  |  |  | 0.1 | 0.0, 0.9 |  |  | | 4.5 | 3.0, 6.6 |  |  | 11.7 | 6.7, 19.5 |
| BP, blood pressure; FPG, fasting plasma glucose; HDL-C, high density lipoprotein cholesterol; TG, triglyceride, WC, Waist circumference. | | | | | | | | | | | | | | |
|  | | | | | | | | | | | | | | |
