# Supplementary material for: Cardiometabolic risk profiles in a Sri Lankan twin and singleton sample
Source: PLoS One. 2022 Nov 7;17(11):e0276647. doi: 10.1371/journal.pone.0276647 (PMC9639827; doi:10.1371/journal.pone.0276647)
Supplement: S3 Table — (DOCX) [file pone.0276647.s003.docx]

**S3 Table. Description of latent cardiometabolic classes by metabolic syndrome components contributing to the model in women (N=1967)**

|  | **Healthy values** |  | **Class 1**  Healthy, WC (53.1%) | | |  | **Class 2**  Obese, HDLC, Treated BP, FPG (32.8%) | | |  | **Class 3**  WC, Diabetes (7.2%) | | |  | **Class 4**  WC, Untreated BP, FPG (6.8%) | | |
| --- | --- | --- | --- | --- | --- | --- | --- | --- | --- | --- | --- | --- | --- | --- | --- | --- | --- |
|  |  |  | Mean | % | 95% CI |  | Mean | % | 95% CI |  | Mean | % | 95% CI |  | Mean | % | 95% CI |
| WC (cm) | <80.0 |  | 85.6 |  | 84.9, 86.3 |  | 98.3 |  | 97.5, 99.1 |  | 95.9 |  | 94.2, 97.6 |  | 91.4 |  | 89.3, 93.4 |
| TG (mmol/L) | <1.7 |  | 1.0 |  | 0.9, 1.0 |  | 1.7 |  | 1.6, 1.7 |  | 1.7 |  | 1.6, 1.8 |  | 1.3 |  | 1.2, 1.5 |
| HDL-C (mmol/L) | >1.29 |  | 1.4 |  | 1.3, 1.4 |  | 1.2 |  | 1.2, 1.3 |  | 1.4 |  | 1.3, 1.4 |  | 1.5 |  | 1.4, 1.5 |
| Systolic BP (mm Hg) | <130.0 |  | 105.9 |  | 105.2, 106.7 |  | 122.0 |  | 120.7, 123.2 |  | 126.1 |  | 122.5, 129.7 |  | 158.3 |  | 154.7, 161.8 |
| Diastolic BP (mm Hg) | <85.0 |  | 71.4 |  | 70.8, 71.9 |  | 81.5 |  | 80.6, 82.3 |  | 81.5 |  | 79.7, 83.3 |  | 86.8 |  | 84.6, 89.1 |
| FPG (mmol/L) | <5.6 |  | 5.1 |  | 5.1, 5.1 |  | 5.9 |  | 5.8, 6.0 |  | 13.3 |  | 12.7, 13.9 |  | 6.0 |  | 5.8, 6.2 |
| Diabetes |  |  |  | 1.2 | 0.7, 2.0 |  |  | 16.1 | 13.5, 19.2 |  |  | 69.7 | 61.8, 76.6 |  |  | 33.3 | 25.7, 41.9 |
| Blood pressure medication |  |  |  | 0.4 | 0.1, 1.00 |  |  | 59.4 | 50.5, 67.7 |  |  | 25.6 | 22.3, 29.3 |  |  | 27.7 | 20.6, 36.1 |
| Cholesterol medication |  |  |  | 0.5 | 0.2, 1.2 |  |  | 10.0 | 7.8, 12.9 |  |  | 9.9 | 5.8, 16.4 |  |  | 15.9 | 10.3, 23.9 |
| BP, blood pressure; FPG, fasting plasma glucose; HDL-C, high density lipoprotein cholesterol; TG, triglyceride, WC, Waist circumference. | | | | | | | | | | | | | | | | | |
|  | | | | | | | | | | | | | | | | | |
