# Supplementary material for: Cardiometabolic risk profiles in a Sri Lankan twin and singleton sample
Source: PLoS One. 2022 Nov 7;17(11):e0276647. doi: 10.1371/journal.pone.0276647 (PMC9639827; doi:10.1371/journal.pone.0276647)
Supplement: S4 Table — (DOCX) [file pone.0276647.s004.docx]

S4 Table. Prevalence distribution and unadjusted associations of sociodemographic characteristics and health behaviours with latent classes in men (N=1509)

|  | **Prevalence distribution** | | | | | | | | | | |  | **Unadjusted regression analysis** | | | | | | |
| --- | --- | --- | --- | --- | --- | --- | --- | --- | --- | --- | --- | --- | --- | --- | --- | --- | --- | --- | --- |
|  | **Class 1**  Healthy (52.3%) | | |  | **Class 2**  WC, TG, FPG (40.2%) | | |  | **Class 3**  WC, TG, Diabetes (7.6%) | | |  | **Class 1**  Healthy (52.3%) |  | **Class 2**  WC, TG, FPG (40.2%) | |  | **Class 3**  WC, TG, Diabetes (7.6%) | |
|  | **No.** | **Mean** | **%** |  | **No.** | **Mean** | **%** |  | **No.** | **Mean** | **%** |  | **OR** |  | **OR** | **95% CI** |  | **OR** | **95% CI** |
| Age (years) | 789 | 38.0 |  |  | 606 | 45.5 |  |  | 114 | 51.5 |  |  | 1.00 |  | 1.04 | 1.03, 1.05 |  | 1.07 | 1.05, 1.08 |
| Marital status |  |  |  |  |  |  |  |  |  |  |  |  |  |  |  |  |  |  |  |
| Married | 501 |  | 64.5 |  | 493 |  | 81.9 |  | 102 |  | 91.1 |  |  |  | 1.00 |  |  | 1.00 |  |
| Not married | 276 |  | 35.5 |  | 109 |  | 18.1 |  | 10 |  | 8.9 |  | 1.00 |  | 0.40 | 0.31, 0.53 |  | 0.18 | 0.09, 0.35 |
| Ethnic minority |  |  |  |  |  |  |  |  |  |  |  |  |  |  |  |  |  |  |  |
| Sinhala | 725 |  | 93.4 |  | 95.5 |  | 91.2 |  | 102 |  | 91.1 |  |  |  | 1.00 |  |  | 1.00 |  |
| Ethnic minority | 51 |  | 6.6 |  | 38 |  | 6.3 |  | 10 |  | 8.9 |  | 1.00 |  | 0.96 | 0.61, 1.51 |  | 1.39 | 0.67, 2.90 |
| Education |  |  |  |  |  |  |  |  |  |  |  |  |  |  |  |  |  |  |  |
| ≤ Grade 5 | 59 |  | 7.6 |  | 33 |  | 5.5 |  | 5 |  | 4.5 |  | 1.00 |  | 0.61 | 0.37, 0.99 |  | 0.56 | 0.21, 1.51 |
| Grade 6 - O/Ls | 502 |  | 64.9 |  | 370 |  | 61.9 |  | 75 |  | 67.0 |  | 1.00 |  | 0.80 | 0.62, 1.03 |  | 0.99 | 0.63, 1.55 |
| ≥ A/Ls | 212 |  | 27.4 |  | 195 |  | 32.6 |  | 32 |  | 28.6 |  |  |  | 1.00 |  |  | 1.00 |  |
| Occupational class |  |  |  |  |  |  |  |  |  |  |  |  |  |  |  |  |  |  |  |
| Managers/Professionals | 64 |  | 8.3 |  | 59 |  | 9.8 |  | 13 |  | 11.8 |  |  |  | 1.00 |  |  | 1.00 |  |
| Skilled manual/non-manual workers | 476 |  | 61.8 |  | 366 |  | 60.9 |  | 60 |  | 54.5 |  | 1.00 |  | 0.83 | 0.56, 1.24 |  | 0.62 | 0.32, 1.20 |
| Elementary occupations | 136 |  | 17.7 |  | 49 |  | 8.2 |  | 8 |  | 7.3 |  | 1.00 |  | 0.39 | 0.24, 0.64 |  | 0.29 | 0.11, 0.74 |
| Not in employment | 94 |  | 12.2 |  | 127 |  | 21.1 |  | 29 |  | 26.4 |  | 1.00 |  | 1.47 | 0.92, 2.33 |  | 1.52 | 0.73, 3.16 |
| Financial strain |  |  |  |  |  |  |  |  |  |  |  |  |  |  |  |  |  |  |  |
| Low | 595 |  | 76.6 |  | 490 |  | 81.4 |  | 87 |  | 77.7 |  |  |  | 1.00 |  |  | 1.00 |  |
| Moderate | 117 |  | 15.1 |  | 61 |  | 10.1 |  | 14 |  | 12.5 |  | 1.00 |  | 0.63 | 0.45, 0.89 |  | 0.82 | 0.45, 1.50 |
| High | 65 |  | 8.4 |  | 51 |  | 8.5 |  | 11 |  | 9.8 |  | 1.00 |  | 0.95 | 0.64, 1.41 |  | 1.16 | 0.59, 2.27 |
| Physical activity |  |  |  |  |  |  |  |  |  |  |  |  |  |  |  |  |  |  |  |
| Low | 59 |  | 7.6 |  | 56 |  | 9.3 |  | 12 |  | 10.9 |  | 1.00 |  | 1.45 | 0.98, 2.15 |  | 2.12 | 0.95, 3.61 |
| Moderate | 159 |  | 20.6 |  | 180 |  | 30.1 |  | 37 |  | 33.6 |  | 1.00 |  | 1.73 | 1.34, 2.24 |  | 2.12 | 1.35, 3.33 |
| High | 55 |  | 71.8 |  | 363 |  | 60.6 |  | 61 |  | 55.5 |  |  |  | 1.00 |  |  | 1.00 |  |
| Diet risk score^a^ | 777 |  | 2.0 |  | 601 |  | 1.8 |  | 112 |  | 1.7 |  | 1.00 |  | 0.89 | 0.80, 0.99 |  | 0.81 | 0.67, 0.99 |
| Alcohol use |  |  |  |  |  |  |  |  |  |  |  |  |  |  |  |  |  |  |  |
| No misuse | 564 |  | 72.7 |  | 412 |  | 68.6 |  | 73 |  | 65.2 |  |  |  | 1.00 |  |  | 1.00 |  |
| Hazardous use | 157 |  | 20.2 |  | 157 |  | 26.1 |  | 28 |  | 25.0 |  | 1.00 |  | 1.37 | 1.06, 1.78 |  | 1.38 | 0.85, 2.24 |
| Harmful use | 55 |  | 7.1 |  | 32 |  | 5.3 |  | 11 |  | 9.8 |  | 1.00 |  | 0.80 | 0.50, 1.26 |  | 1.55 | 0.78, 3.08 |
| Smoking |  |  |  |  |  |  |  |  |  |  |  |  |  |  |  |  |  |  |  |
| Never smoked | 463 |  | 8.7 |  | 351 |  | 57.9 |  | 62 |  | 54.4 |  |  |  |  | 1.00 |  | 1.00 |  |
| Ex /occasional smoker | 134 |  | 17.0 |  | 147 |  | 24.3 |  | 24 |  | 21.1 |  | 1.00 |  | 1.45 | 1.09, 1.92 |  | 1.34 | 0.79, 2.25 |
| Current daily smoker | 192 |  | 24.3 |  | 108 |  | 17.8 |  | 28 |  | 24.6 |  | 1.00 |  | 0.74 | 0.56, 0.99 |  | 1.09 | 0.67, 1.77 |
| ^a^ Diet risk score is on a scale from 0-5, where higher scores indicate poorer diet.  BP, blood pressure; FPG, fasting plasma glucose; HDL-C, high density lipoprotein cholesterol; TG, triglyceride; WC, waist circumference. | | | | | | | | | | | | | | | | | | | |
