# Supplementary material for: Cardiometabolic risk profiles in a Sri Lankan twin and singleton sample
Source: PLoS One. 2022 Nov 7;17(11):e0276647. doi: 10.1371/journal.pone.0276647 (PMC9639827; doi:10.1371/journal.pone.0276647)
Supplement: S5 Table — (DOCX) [file pone.0276647.s005.docx]

S5 Table. Prevalence distribution and unadjusted associations of sociodemographic characteristics and health behaviours with latent classes in women (N=1967)

|  | **Prevalence distribution** | | | | | | | | | | | | | | |  | **Unadjusted regression analysis** | | | | | | | | | |  |
| --- | --- | --- | --- | --- | --- | --- | --- | --- | --- | --- | --- | --- | --- | --- | --- | --- | --- | --- | --- | --- | --- | --- | --- | --- | --- | --- | --- |
|  | **Class 1**  Healthy, WC (53.1%) | | |  | **Class 2**  Obese, HDLC, Treated BP, FPG (32.8%) | | |  | **Class 3**  WC, Diabetes (7.2%) | | |  | **Class 4**  WC, Untreated BP, FPG (6.8%) | | |  | **Class 1**  Healthy, WC (53.1%) |  | **Class 2**  Obese, HDLC, Treated BP, FPG (32.8%) | |  | **Class 3**  WC, Diabetes (7.2%) | |  | **Class 4**  WC, Untreated BP, FPG (6.8%) | |  |
|  | **No.** | **Mean** | **%** |  | **No.** | **Mean** | **%** |  | **No.** | **Mean** | **%** |  | **No.** | **Mean** | **%** |  | **OR** |  | **OR** | **95% CI** |  | **OR** | **95% CI** |  | **OR** | **95% CI** | |
| Age (years) | 1046 | 36.3 |  |  | 646 | 48.2 |  |  | 142 | 50.1 |  |  | 133 | 66.1 |  |  | 1.00 |  | 1.09 | 1.08, 1.10 |  | 1.10 | 1.09, 1.12 |  | 1.23 | 1.20, 1.26 | |
| Marital status |  |  |  |  |  |  |  |  |  |  |  |  |  |  |  |  |  |  |  |  |  |  |  |  |  |  | |
| Married | 722 |  | 69.8 |  | 505 |  | 78.4 |  | 119 |  | 83.8 |  | 71 |  | 53.8 |  |  |  | 1.00 |  |  | 1.00 |  |  |  | 1.00 | |
| Not married | 313 |  | 30.2 |  | 139 |  | 21.6 |  | 23 |  | 16.2 |  | 61 |  | 46.2 |  | 1.00 |  | 0.63 | 0.50, 0.80 |  | 0.45 | 0.28, 0.71 |  | 1.98 | 1.36, 2.90 | |
| Ethnic minority |  |  |  |  |  |  |  |  |  |  |  |  |  |  |  |  |  |  |  |  |  |  |  |  |  |  | |
| Sinhala | 956 |  | 92.4 |  | 592 |  | 91.9 |  | 129 |  | 90.8 |  | 129 |  | 97.7 |  |  |  | 1.00 |  |  | 1.00 |  |  | 1.00 |  | |
| Ethnic minority | 79 |  | 7.6 |  | 52 |  | 8.1 |  | 13 |  | 9.2 |  | 3 |  | 2.3 |  | 1.00 |  | 1.06 | 0.72, 1.58 |  | 1.22 | 0.62, 2.38 |  | 0.28 | 0.09, 0.92 | |
| Education |  |  |  |  |  |  |  |  |  |  |  |  |  |  |  |  |  |  |  |  |  |  |  |  |  |  | |
| ≤ Grade 5 | 64 |  | 6.2 |  | 78 |  | 12.2 |  | 13 |  | 9.2 |  | 23 |  | 17.4 |  | 1.00 |  | 2.80 | 1.88, 4.15 |  | 2.30 | 1.07, 4.98 |  | 7.91 | 3.86, 16.19 | |
| Grade 6 , O/Ls | 594 |  | 57.6 |  | 398 |  | 62.3 |  | 95 |  | 67.4 |  | 92 |  | 69.7 |  | 1.00 |  | 1.54 | 1.21, 1.95 |  | 1.81 | 1.17, 2.80 |  | 3.41 | 1.94, 5.98 | |
| ≥ A/Ls | 374 |  | 36.2 |  | 163 |  | 25.5 |  | 33 |  | 23.4 |  | 17 |  | 12.9 |  |  |  | 1.00 |  |  | 1.00 |  |  | 1.00 |  | |
| Occupational class |  |  |  |  |  |  |  |  |  |  |  |  |  |  |  |  |  |  |  |  |  |  |  |  |  |  | |
| Managers/Professionals | 86 |  | 8.4 |  | 34 |  | 5.3 |  | 7 |  | 5.0 |  | 3 |  | 2.3 |  |  |  | 1.00 |  |  | 1.00 |  |  | 1.00 |  | |
| Skilled manual/non, manual workers | 299 |  | 29.1 |  | 141 |  | 22.1 |  | 30 |  | 21.4 |  | 11 |  | 8.3 |  | 1.00 |  | 1.19 | 0.74, 1.92 |  | 1.23 | 0.53, 2.87 |  | 1.05 | 0.29, 3.89 | |
| Elementary occupations | 51 |  | 5.0 |  | 29 |  | 4.5 |  | 10 |  | 7.1 |  | 5 |  | 3.8 |  | 1.00 |  | 1.44 | 0.76, 2.72 |  | 2.41 | 0.86, 6.75 |  | 2.81 | 0.64, 12.36 | |
| Not in employment | 593 |  | 57.6 |  | 434 |  | 68.0 |  | 93 |  | 66.4 |  | 113 |  | 85.6 |  | 1.00 |  | 1.85 | 1.18, 2.92 |  | 1.93 | 0.86, 4.31 |  | 5.46 | 1.70, 17.60 | |
| Financial strain |  |  |  |  |  |  |  |  |  |  |  |  |  |  |  |  |  |  |  |  |  |  |  |  |  |  | |
| Low | 784 |  | 75.8 |  | 453 |  | 70.3 |  | 94 |  | 66.2 |  | 87 |  | 65.9 |  |  |  | 1.00 |  |  | 1.00 |  |  | 1.00 |  | |
| Moderate | 136 |  | 13.2 |  | 108 |  | 16.8 |  | 26 |  | 18.3 |  | 23 |  | 17.4 |  | 1.00 |  | 1.37 | 1.03, 1.83 |  | 1.59 | 0.99, 2.57 |  | 1.52 | 0.91, 2.55 | |
| High | 114 |  | 11.0 |  | 83 |  | 12.9 |  | 22 |  | 15.5 |  | 22 |  | 16.7 |  | 1.00 |  | 1.26 | 0.92, 1.72 |  | 1.61 | 0.97, 2.68 |  | 1.74 | 1.04, 2.90 | |
| Physical activity |  |  |  |  |  |  |  |  |  |  |  |  |  |  |  |  |  |  |  |  |  |  |  |  |  |  | |
| Low | 79 |  | 7.7 |  | 36 |  | 5.6 |  | 11 |  | 7.7 |  | 13 |  | 9.8 |  | 1.00 |  | 0.66 | 0.43, 1.01 |  | 0.92 | 0.45, 1.87 |  | 1.34 | 0.68, 2.63 | |
| Moderate | 255 |  | 24.9 |  | 129 |  | 20.1 |  | 26 |  | 18.3 |  | 34 |  | 25.8 |  | 1.00 |  | 0.74 | 0.57, 0.94 |  | 0.67 | 0.42, 1.07 |  | 1.09 | 0.70, 1.68 | |
| High | 692 |  | 67.4 |  | 476 |  | 74.3 |  | 105 |  | 73.9 |  | 85 |  | 64.4 |  |  |  | 1.00 |  |  | 1.00 |  |  | 1.00 |  | |
| Diet risk score^a^ | 1034 |  | 1.8 |  | 644 |  | 1.7 |  | 142 |  | 1.6 |  | 132 |  | 1.5 |  | 1.00 |  | 0.88 | 0.79, 0.97 |  | 0.80 | 0.67, 0.95 |  | 0.76 | 0.64, 0.90 | |
| ^a^ Diet risk score is on a scale from 0, 5, where higher scores indicate poorer diet.  BP, blood pressure; FPG, fasting plasma glucose; HDL, C, high density lipoprotein cholesterol; TG, triglyceride; WC, waist circumference. | | | | | | | | | | | | | | | | | | | | | | | | | | | |
