# Supplementary material for: Cardiometabolic risk profiles in a Sri Lankan twin and singleton sample
Source: PLoS One. 2022 Nov 7;17(11):e0276647. doi: 10.1371/journal.pone.0276647 (PMC9639827; doi:10.1371/journal.pone.0276647)
Supplement: S6 Table — (DOCX) [file pone.0276647.s006.docx]

S6 Table. Description of latent cardiometabolic classes according to other cardiometabolic risk factors in men (N=1509)

|  |  | **Class 1**  Healthy, WC  (53.1%) | | | |  | **Class 2**  Obese, HDLC, Treated BP, FPG (32.8%) | | |  | **Class 3**  WC, Diabetes  (7.2%) | | |
| --- | --- | --- | --- | --- | --- | --- | --- | --- | --- | --- | --- | --- | --- |
|  | **Normal values** |  | **Mean** | **%** | **95% CI** |  | **Mean** | **%** | **95% CI** |  | **Mean** | **%** | **95% CI** |
| BMI (kg/m^2^) | <23.0^a^ |  | 20.9 |  | 20.7, 21.1 |  | 25.4 |  | 25.1, 25.8 |  | 25.2 |  | 24.5, 26.0 |
| Total cholesterol (mmol/L) | <5.18 |  | 5.10 |  | 5.02, 5.18 |  | 5.24 |  | 5.14, 5.33 |  | 5.17 |  | 4.96, 5.38 |
| Ratio of total cholesterol /HDL-C | <6.0 |  | 4.12 |  | 4.05, 4.20 |  | 4.73 |  | 4.63, 4.83 |  | 4.48 |  | 4.28, 4.68 |
| LDL-C (mmol/L) | <4.1 |  | 3.21 |  | 3.14, 3.28 |  | 3.22 |  | 3.13, 3.30 |  | 3.11 |  | 2.94, 3.29 |
| VLDL-C (mmol/L) |  |  | 0.62 |  | 0.60, 0.64 |  | 0.88 |  | 0.84, 0.92 |  | 0.87 |  | 0.79, 0.94 |
| HbA1c (mean %) | <6.0 |  | 5.4 |  | 5.4, 5.4 |  | 5.8 |  | 5.8, 5.9 |  | 9.4 |  | 9.1, 9.7 |
| Insulin resistance (HOMA-IR score) | <2.0 |  | 1.16 |  | 1.11, 1.22 |  | 2.20 |  | 2.09, 2.31 |  | 2.65 |  | 2.36, 2.95 |
| Insulin (pmol/L) | <174 |  | 61.9 |  | 58.7, 65.0 |  | 117.1 |  | 111.1, 123.1 |  | 116.0 |  | 102.5, 129.5 |
| SGOT (U/L) | 10-30 |  | 28.1 |  | 26.8, 29.4 |  | 30.2 |  | 29.2, 31.2 |  | 29.0 |  | 26.1, 32.0 |
| Serum creatinine (mg/dL) | <1.2 |  | 0.96 |  | 0.95, 0.97 |  | 1.01 |  | 0.99, 1.03 |  | 0.95 |  | 0.92, 0.99 |
| Urine microalbumin (mg/L) | <30 |  | 14.2 |  | 12.9, 15.5 |  | 23.2 |  | 18.6, 27.7 |  | 65.6 |  | 25.8, 105.4 |
| ACR | <30 |  | 12.6 |  | 11.4, 13.7 |  | 30.5 |  | 18.5, 42.6 |  | 130.1 |  | 41.0, 219.3 |
| CRP (mg/L) | <3.1 |  | 2.0 |  | 1.7, 2.3 |  | 3.2 |  | 2.7, 3.6 |  | 4.3 |  | 3.0, 5.6 |
| Self-reported heart condition | - |  |  | 10.7 | 8.6, 13.2 |  |  | 15.6 | 13.0, 18.7 |  |  | 14.3 | 8.9, 22.1 |
| Self-reported hypertension | - |  |  | 1.9 | 1.2, 3.2 |  |  | 22.9 | 19.6, 26.6 |  |  | 29.5 | 21.6, 38.8 |
| ^a^ In line with WHO recommendations for South Asian populations  ACR, Urine Albumin to Creatinine Ratio; BMI, body mass index; CRP, C-reactive protein; HbA1c, Hemoglobin A1C; HOMA-IR, Homeostatic Model Assessment of Insulin Resistance; LCL-C, low-density lipoprotein cholesterol, SGOT, serum glutamic-oxaloacetic transaminase; VLCL-C, very low-density lipoprotein cholesterol | | | | | | | | | | | | | |
