# Supplementary material for: Cardiometabolic risk profiles in a Sri Lankan twin and singleton sample
Source: PLoS One. 2022 Nov 7;17(11):e0276647. doi: 10.1371/journal.pone.0276647 (PMC9639827; doi:10.1371/journal.pone.0276647)
Supplement: S7 Table — (DOCX) [file pone.0276647.s007.docx]

S7 Table. Description of latent cardiometabolic classes according to other cardiometabolic risk factors in women (N=1967)

|  |  |  | **Class 1**  Healthy, WC (53.1%) | | |  | **Class 2**  Obese, HDLC, Treated BP, FPG (32.8%) | | |  | **Class 3**  WC, Diabetes (7.2%) | | |  | **Class 4**  WC, Untreated BP, FPG (6.8%) | | |
| --- | --- | --- | --- | --- | --- | --- | --- | --- | --- | --- | --- | --- | --- | --- | --- | --- | --- |
|  | **Normal values** |  | **Mean** | **%** | **95% CI** |  | **Mean** | **%** | **95% CI** |  | **Mean** | **%** | **95% CI** |  | **Mean** | **%** | **95% CI** |
| BMI (kg/m^2^) | <23.0^a^ |  | 22.7 |  | 22.4, 23.0 |  | 27.3 |  | 27.0, 27.7 |  | 26.3 |  | 25.5, 27.1 |  | 23.4 |  | 22.6, 24.1 |
| Total cholesterol (mmol/L) | <5.18 |  | 4.95 |  | 4.89, 5.02 |  | 5.43 |  | 5.34, 5.52 |  | 5.67 |  | 5.49, 5.85 |  | 5.42 |  | 5.22, 5.62 |
| Ratio of total cholesterol /HDL-C | <6.0 |  | 3.73 |  | 3.68, 3.78 |  | 4.49 |  | 4.40, 4.58 |  | 4.31 |  | 4.14, 4.48 |  | 3.81 |  | 3.63, 3.98 |
| LDL-C (mmol/L) | <4.1 |  | 3.16 |  | 3.11, 3.22 |  | 3.44 |  | 3.36, 3.52 |  | 3.55 |  | 3.38, 3.71 |  | 3.34 |  | 3.16, 3.52 |
| VLDL-C (mmol/L) |  |  | 0.44 |  | 0.43, 0.45 |  | 0.75 |  | 0.72, 0.78 |  | 0.78 |  | 0.73, 0.83 |  | 0.62 |  | 0.57, 0.67 |
| HbA1c (mean %) | <6.0 |  | 5.4 |  | 5.4, 5.5 |  | 6.0 |  | 6.0, 6.1 |  | 10.2 |  | 9.9, 10.5 |  | 6.3 |  | 6.1, 6.6 |
| Insulin resistance (HOMA-IR score) | <2.0 |  | 1.34 |  | 1.29, 1.39 |  | 2.14 |  | 2.05, 2.24 |  | 3.26 |  | 2.43, 4.10 |  | 1.56 |  | 1.37, 1.75 |
| Insulin (pmol/L) | <174 |  | 72.1 |  | 69.2, 74.9 |  | 112.8 |  | 107.7, 118.0 |  | 108.2 |  | 98.1, 118.3 |  | 82.3 |  | 71.9, 92.8 |
| SGOT (U/L) | 10-30 |  | 20.8 |  | 20.3, 21.3 |  | 24.3 |  | 23.4, 25.2 |  | 26.4 |  | 23.1, 29.7 |  | 24.1 |  | 22.2, 26.0 |
| Serum creatinine (mg/dL) | <1.2 |  | 0.72 |  | 0.71, 0.73 |  | 0.74 |  | 0.73, 0.75 |  | 0.72 |  | 0.69, 0.74 |  | 0.90 |  | 0.77, 1.03 |
| Urine microalbumin (mg/L) | <30 |  | 13.2 |  | 12.0, 14.3 |  | 18.2 |  | 13.5, 22.8 |  | 36.0 |  | 17.7, 54.3 |  | 26.1 |  | 10.9, 41.3 |
| ACR | <30 |  | 17.5 |  | 15.8, 19.2 |  | 24.3 |  | 20.7, 28.0 |  | 134.6 |  | 32.1, 237.2 |  | 96.0 |  | 0, 192.4 |
| CRP (mg/L) | <3.1 |  | 2.6 |  | 2.3, 2.8 |  | 4.3 |  | 3.9, 4.8 |  | 7.1 |  | 5.6, 8.6 |  | 2.8 |  | 2.1, 3.5 |
| Self-reported heart condition | - |  |  | 11.3 | 9.5, 13.5 |  |  | 17.5 | 14.8, 20.7 |  |  | 17.6 | 12.1, 25.0 |  |  | 17.4 | 11.7, 25.2 |
| Self-reported hypertension | - |  |  | 3.6 | 2.6, 4.9 |  |  | 28.7 | 25.3, 32.5 |  |  | 29.6 | 22.5, 37.8 |  |  | 53.8 | 44.9, 62.4 |
| ^a^ In line with WHO recommendations for South Asian populations  ACR, Urine Albumin to Creatinine Ratio; BMI, body mass index; CRP, C-reactive protein; HbA1c, Hemoglobin A1C; HOMA-IR, Homeostatic Model Assessment of Insulin Resistance; LCL-C, low-density lipoprotein cholesterol, SGOT, serum glutamic-oxaloacetic transaminase; VLCL-C, very low-density lipoprotein cholesterol | | | | | | | | | | | | | | | | | |
